# Impact of COVID-19 on autism spectrum disorder management - a therapist perspective

**DOI:** 10.1192/j.eurpsy.2021.301

**Published:** 2021-08-13

**Authors:** H. Atturu, S. Lakhani

**Affiliations:** 1 Department Of Psychiatry, CARE Hospitals, Hyderabad, India; 2 Department Of Special Education, Roshini Counselling Centre, Hyderabad, India

**Keywords:** autism spectrum disorder, Digital based therapy, COVID-19, online therapy

## Abstract

**Introduction:**

Therapist led interventions form a core element in the management of children with Autism Spectrum Disorder (ASD) in India. COVID-19 pandemic has disrupted several aspects of ASD management.

**Objectives:**

This study aims to understand the impact of COVID-19 pandemic on ASD therapies from a therapist perspective.

**Methods:**

An online survey was conducted using a google form questionnaire disseminated among ASD therapists. The form was open for response between 23^rd^ of June and 23^rd^ of July 2020. The responses were extracted into an excel sheet and analysed using descriptive statistics.

**Results:**

41 out of 75 therapists with mean age of 44 years (21 – 58 years) responded to the survey. 48% were women. Majority of them were either special educators (49%) or ASD therapists (32%) with professional experience of >5 years (63%). Majority of the therapists felt that there is significant disruption during the pandemic with reduction in conventional therapies (63% to 17%) and increase in online therapies (15% to 61%). They also felt that this disruption had moderate to severe impact on child’s learning (73%) and parents emotional and psychological well being (85%). Only 22% of therapists were using digital based therapy (DBT) before the pandemic. Although 51% of the therapists were not entirely sure whether DBT augments parents and therapists’ efforts, majority (65%) were willing to use them.

**Conclusions:**

COVID-19 pandemic has significantly disrupted ASD therapy in India. Willingness to use online and digital based therapies could open up a new dimension. Reliable and effective Artificial-Intelligence based therapies are the need of the day.

**Disclosure:**

Medical advisor for CognitiveBotics

